# Potentiation of natural killer cells to overcome cancer resistance to NK cell-based therapy and to enhance antibody-based immunotherapy

**DOI:** 10.3389/fimmu.2023.1275904

**Published:** 2023-11-24

**Authors:** Massimo Fantini, Philip Martin Arlen, Kwong Yok Tsang

**Affiliations:** Precision Biologics, Inc., Bethesda, MD, United States

**Keywords:** NK cells, ADCC, modulatory cytokines, adoptive NK cell therapy, monoclonal antibody, CAR-NK

## Abstract

Natural killer (NK) cells are cellular components of the innate immune system that can recognize and suppress the proliferation of cancer cells. NK cells can eliminate cancer cells through direct lysis, by secreting perforin and granzymes, or through antibody-dependent cell-mediated cytotoxicity (ADCC). ADCC involves the binding of the Fc gamma receptor IIIa (CD16), present on NK cells, to the constant region of an antibody already bound to cancer cells. Cancer cells use several mechanisms to evade antitumor activity of NK cells, including the accumulation of inhibitory cytokines, recruitment and expansion of immune suppressor cells such as myeloid-derived suppressor cells (MDSCs) and regulatory T cells (Tregs), modulation of ligands for NK cells receptors. Several strategies have been developed to enhance the antitumor activity of NK cells with the goal of overcoming cancer cells resistance to NK cells. The three main strategies to engineer and boost NK cells cytotoxicity include boosting NK cells with modulatory cytokines, adoptive NK cell therapy, and the employment of engineered NK cells to enhance antibody-based immunotherapy. Although the first two strategies improved the efficacy of NK cell-based therapy, there are still some limitations, including immune-related adverse events, induction of immune-suppressive cells and further cancer resistance to NK cell killing. One strategy to overcome these issues is the combination of monoclonal antibodies (mAbs) that mediate ADCC and engineered NK cells with potentiated anti-cancer activity. The advantage of using mAbs with ADCC activity is that they can activate NK cells, but also favor the accumulation of immune effector cells to the tumor microenvironment (TME). Several clinical trials reported that combining engineered NK cells with mAbs with ADCC activity can result in a superior clinical response compared to mAbs alone. Next generation of clinical trials, employing engineered NK cells with mAbs with higher affinity for CD16 expressed on NK cells, will provide more effective and higher-quality treatments to cancer patients.

## 1 Introduction

Cancer is considered one of the deadliest diseases in the world. In 2020, cancer caused 10 million cancer deaths worldwide, with an estimate of 19.3 million new cases (1). Surgery, radiotherapy and chemotherapy are still considered as principal treatment options in clinical oncology. Unfortunately, especially in the case of radiotherapy and chemotherapy, their employment can cause severe side effects that do not allow patients to complete the treatment, with a consequent failure in controlling tumor growth and metastasis (2, 3).

In the last few decades, more selective and less toxic therapies have been developed. Among them, immunotherapy represents a novel and very promising approach for the treatment of cancer patients. Immunotherapy enhances the capacity of immune system to attack and eliminate cancer cells in a more selective manner with a reduced toxicity towards normal tissues (4).

The immune system targets and counteracts the proliferation of malignant cells through the immunoediting process, that is composed of three phases: elimination, equilibrium, and escape (5).

*Elimination*: in this phase, normal cells are transformed into cancer cells through the process of carcinogenesis. Factors that allow transformed cells to proliferate in an uncontrolled manner during the process of carcinogenesis include upregulation of the expression of oncogenes (genes that regulate cell proliferation) as well as the downregulation of the expression or loss of function of onco-suppressor genes (genes that control cell growth) (6, 7). During the elimination phase, NK cells, macrophages, granulocytes, and cytotoxic T lymphocytes (CTLs) recognize and suppress malignant cells before cancer become clinically visible (5). For example, these immune cells can suppress tumor growth and angiogenesis by releasing interferon (IFN)-γ. NK cells and CTLs can also induce death of cancer cells by apoptosis or can directly lyse them using perforin and granzymes (5, 8, 9).

*Equilibrium*: in this phase, most of cancer cells are in a status of dormancy due to the pressure of the immune system, but some cancer cells can develop mechanisms to resist immune recognition, such as antigen loss, genetic and epigenetic modifications that lead to a defect in antigen presentation, or overexpression of immunosuppressive molecules (5, 9).

*Escape*: in this phase, cancer cells acquire a more aggressive phenotype and the ability to growth without control. These features allow cancer cells to escape from the pressure of immune system and to metastasize to different organs. For example, cancer cells can induce the accumulation of MDSCs in the TME. MDSCs can release immunosuppressive cytokines which in turn can lead to the exhaustion of CTLs. MDSCs can also induce the generation of Tregs. Tregs inhibit antitumor immune response by secreting immune suppressive cytokines (5, 9–12) and by reducing NK cells cytotoxicity in the TME (13).

Potentiate the immune system through cancer immunotherapy can prevent cancer cells to enter in the escape phase of the immunoediting. In this way, the immune system should be able to keep cancer cells permanently in the elimination and/or equilibrium phase (14).

In this review we provide an overview on main cancer immunotherapy strategies based on the stimulation of NK cells, then we describe mAbs that have received FDA approval for the treatment of human cancers with emphasis on their mechanism of action involving NK cells, and finally we focus on strategies to improve antibody-based immunotherapy through the employment of engineered NK cells.

## 2 Role of NK cells in immunoediting and mechanisms of immune-escape

NK cells are one of the cellular components of the innate immune system. These effector cells can target and kill virus-infected cells and cancer cells. They comprise between 5% and 20% of the mononuclear cell fraction in peripheral blood lymphocyte population in humans (15, 16). One of the peculiarities of NK cells is their capacity to kill target cells without requiring their prior sensitization and the expression of major histocompatibility complex (MHC) molecules on cancer cells (17).

In accordance with the CD56 and CD16 status, NK cells can be divided into two subsets. Around 90% of NK cells found in peripheral blood and spleen belongs to the CD56^dim^CD16^+^ subset. These NK cells can kill tumor cells directly or through ADCC, but they produce a low amount of cytokines (17, 18). Conversely, remaining NK cells in peripheral blood and NK cells residing in lymph nodes and tonsils belong to the CD56^bright^CD16^−^ subset. These NK cells have poor ability to kill tumor cell targets but play an important role in modulating immune responses through the secretion of regulatory cytokines, like IFN-γ (17, 18).

NK cells exert their antitumor activity through the binding of their activating receptors, such as NKp46, NKp30, NKp44, NKG2D, and killer cell immunoglobulin-like receptors (KIRs), to NK cells ligands expressed on the surface of cancer cells (19).

One example is represented by the NKG2D/NKG2D ligand (NKG2D-L) pathway (20). NKG2D binds to its ligands expressed on cancer cells, including MICA/B and UL16-binding proteins 1–6 (ULBP1–6) (21).

**Figure 1:**
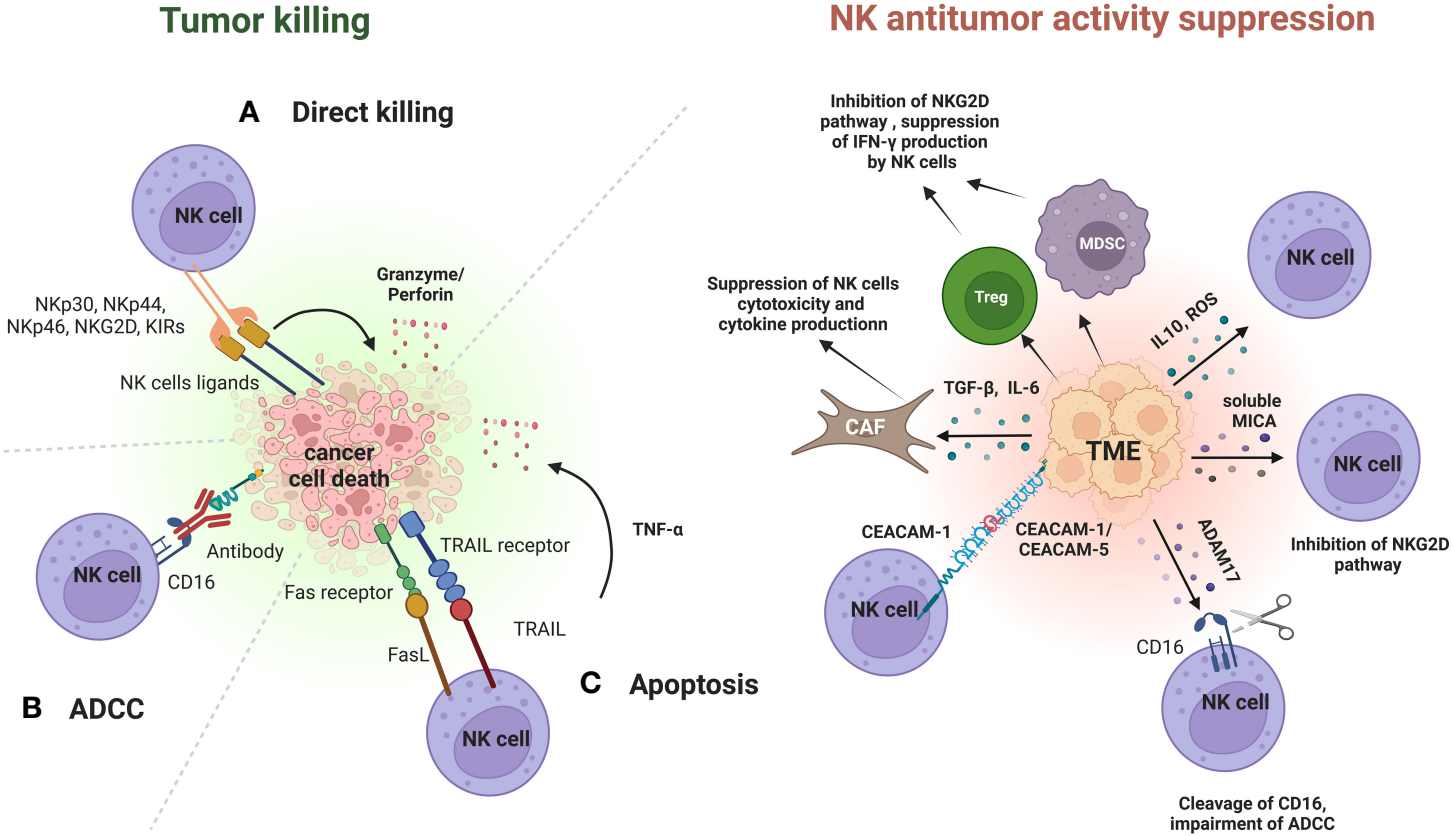
Role of NK cells in immunoediting and mechanisms of immune-escape by cancer cells in the TME. During the elimination phase, NK cells can recognize and suppress cancer cells growth through different mechanisms. After the binding with their ligands, NK cells can kill cancer cells through direct lysis mediated by granzyme and perforin **(A)**, through ADCC **(B)** or through apoptosis mediated by TRAIL and FasL and induced by the release of cytokines (TNF-α) **(C)**. Cancer cells in the TME have developed different strategies to inhibit NK cells antitumor activity. One of these is promotion of recruitment and expansion of Tregs and MDSCs. MDSCs and Tregs can induce NK cells exhaustion and impairment by suppressing INF-γ production, and by inhibiting NKG2D pathway. Furthermore, cancer cells can release TGF-β or IL-6 in the TME to activate cancer-associated fibroblasts (CAFs), that in turn can contribute to the suppression of NK cells cytotoxicity and cytokine production. Other mechanisms include the release of immune suppressive cytokines and of ROS within the TME. Cancer cells can also increase the expression of CEACAM-1 or CEACAM-5 on their surface. These molecules bind to CEACAM-1 expressed on NK cells and this binding can lead to the suppression of NK cells cytotoxicity. Cancer cells can also suppress the NK-mediated ADCC through the proteolytic activity of the enzyme ADAM17. ADAM17 mediates the cleavage of CD16 on NK cells, which in turn suppresses NK cells antitumor activity through ADCC. The immunosuppressive activity of cancer cells towards NK cells is also exerted through the secretion of soluble factors in the bloodstream, such as soluble MICA. Elevated serum levels of soluble MICA suppress NK cell cytotoxicity by affecting the NKG2D pathway. Figure created with BioRender.com.

After the binding with their ligands, NK cells can kill cancer cells through different mechanisms, including direct lysis mediated by perforin, direct killing through death receptor-mediated pathways (i.e. TRAIL and FasL), through the release of cytokines in the TME or through ADCC (22, 23) (**Figure 1**).

Direct lysis through perforin occurs when perforin forms a pore in cell membranes of cancer cells, allowing the release of cytolytic enzymes, such as granzyme A and B, into their cytoplasm which in turn will mediate cell death. Similarly, NK cells can directly kill cancer cells through the binding of TRAIL and FasL to their receptors on cancer cells. This results in caspase activation which in turn mediates cell death by apoptosis (24, 25).

Cytokines released by NK cells have potent antitumor activity. IFN-γ can sensitize cancer cells to CD8+ T-cell killing and polarize macrophage towards M1 state with a stronger antitumoral activity. Tumor necrosis factor-α (TNF-α) can trigger caspase 8-mediated apoptosis of cancer cells and the combination of IFN-γ and TNF-α can induce senescence of cancer cells (23).

NK cells can also mediate killing of cancer cells through ADCC, a mechanism that involves the binding of the constant region (fragment crystallizable, Fc) of an antibody, including mAbs, to the Fc gamma receptor IIIa (FcƴRIIIa, CD16) present on the surface of macrophages and NK cells. This interaction allows NK cells to recognize and kill cancer cells bounded to an antibody (26).

Subjects with head and neck squamous cell carcinoma (HNSCC), oral squamous carcinoma and gastric carcinoma with high levels of NK cells infiltrating their tumors showed an increased survival and a better clinical outcome (27).

Cancer cells in the TME have developed different strategies to inhibit NK cells antitumor activity (**Figure 1**).

One of these is the accumulation of inhibitory cytokines, including IL-10 and TNF-α, that promote the recruitment and expansion of Tregs and MDSCs. MDSCs and Tregs can induce NK cells exhaustion and impairment by suppressing INF-γ production, and by inhibiting NKG2D pathway through membrane-bound TGF-β (27–29).

Furthermore, cancer cells can release TGF-β or IL-6 in the TME to activate cancer-associated fibroblasts (CAFs), that in turn can contribute to the suppression of NK cells cytotoxicity and cytokine production (30).

Other mechanisms include the downregulation of ligands for NK activating receptors, the overexpression of ligands for NK inhibitory receptors, the release of reactive oxygen species (ROS) within the TME (31).

Cancer cells can also increase the expression of carcinoembryonic antigen-related cell adhesion molecule (CEACAM)-1 or CEACAM-5 on their surface. These molecules bind to CEACAM-1 expressed on NK cells and this binding can lead to the suppression of NK cells cytotoxicity (32, 33).

For example, it has been observed that a direct interaction between CEACAM-1 expressed on NK cells and CEACAM-1 expressed on melanoma cells was associated with the poor prognosis of melanoma patients (32).

Cancer cells can also suppress the NK-mediated ADCC by downregulating levels of CD16 on the surface of NK cells through the enhancement of the proteolytic activity of the enzyme ADAM17. ADAM17 is a member of the metalloproteinase superfamily that mediates the cleavage of CD16. The cleavage of CD16 by ADAM17 leads to a decrease of CD16 binding avidity to antibody-coated target cells and in turn can suppress NK cells antitumor activity through ADCC, allowing cancer cells to create an immunosuppressive TME (34).

The immunosuppressive activity of cancer cells towards NK cells is also exerted far away from TME through the secretion of soluble factors in the bloodstream.

One example is represented by the impairment of NK cells antitumor activity by soluble MHC class I polypeptide–related sequence A (MICA) and soluble CEACAM-5. High serum levels of these soluble factors have been associated with cancer progression and metastasis in patients with different types of cancers (35, 36).

Elevated serum levels of soluble MICA have been proved to suppress NK cell cytotoxicity by affecting the NKG2D pathway (37). This phenomenon was associated with a poor prognosis in patients with neuroblastoma (38), hepatocellular carcinoma (39), renal cell carcinoma (40), non-small cell lung cancer (NSCLC) (41), multiple myeloma (MM) (42), pancreatic and colorectal cancer (43, 44).

## 3 NK cells in cancer immunotherapy

NK cell-based immunotherapy represents a promising therapeutic tool for the treatment of solid and hematological tumors.

The three main strategies used to potentiate the antitumor activity of NK cells to overcome the tumor resistance at the level of the TME are boosting NK cells with modulatory cytokines, adoptive NK cell therapy, including CAR-NK cells, and the employment of engineered NK cells to enhance antibody-based immunotherapy.

### 3.1 Enhancement of NK cells antitumor activity through modulatory cytokines

There are several cytokines that are being employed to stimulate the antitumor activity of NK cells.

**Figure 2:**
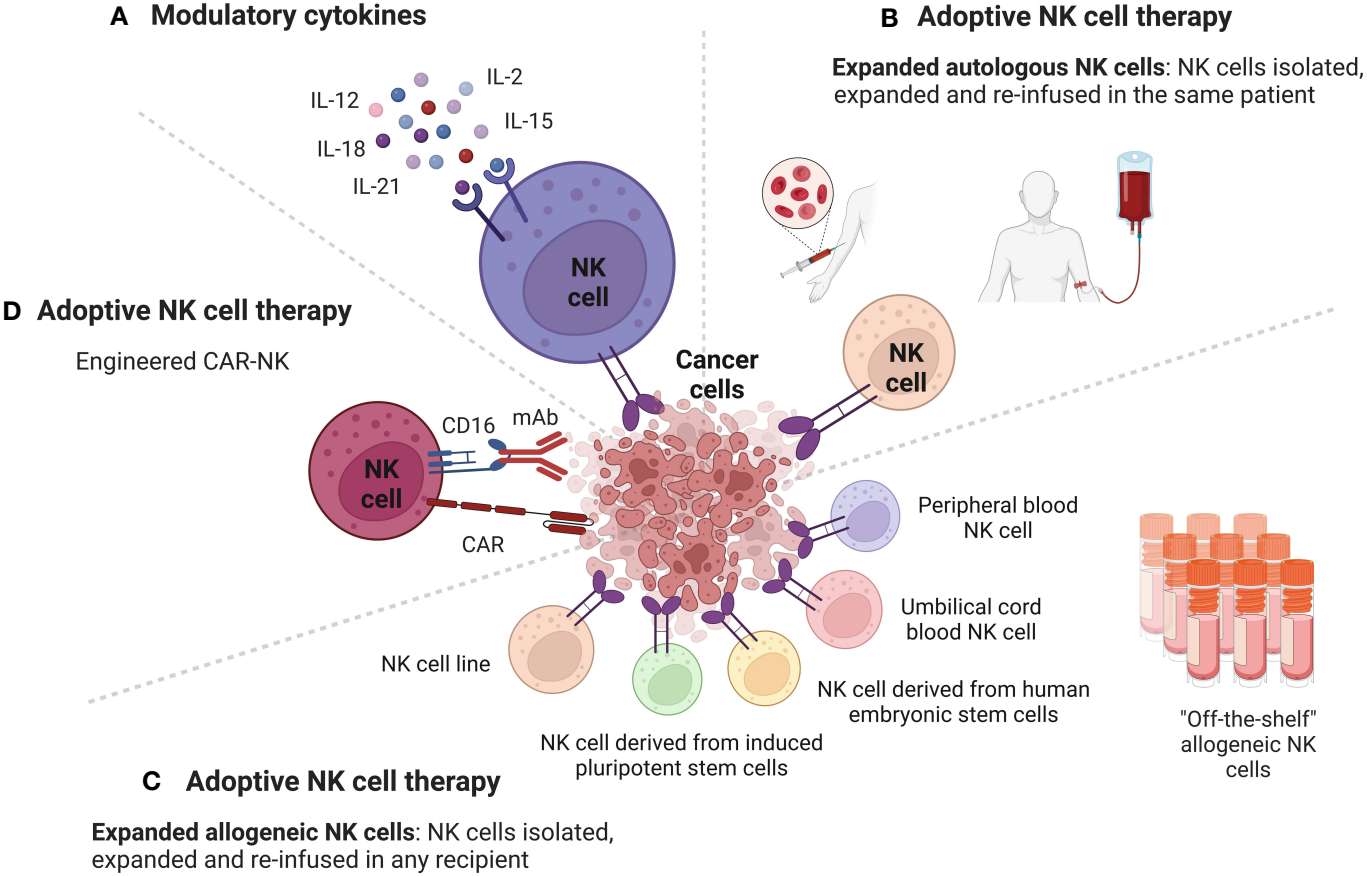
Strategies to potentiate the antitumor activity of NK cells to overcome the tumor resistance at the level of the TME. Cancer cells in the TME have developed several mechanisms to inhibit NK cells antitumor activity. Strategies used to potentiate the antitumor activity of NK cells to overcome the tumor resistance at the level of the TME include boosting NK cells with modulatory cytokines and adoptive NK cell therapy. **(A)** Cytokines, such as IL-2, IL-15, IL-12, IL-18, IL-21 can enhance the anti-cancer activity of NK cells through different mechanisms. NK cells antitumor activity can be also enhanced through the adoptive cell therapy, which consists of isolating NK cells from cellular components of immune system of a subject, engineering them *ex vivo*, expanding their numbers and then re-infuse them in a cancer patient. Sources of NK cells that can be used in adoptive therapy include autologous NK cells, allogeneic NK cells, and CAR-NK cells. **(B)** Adoptive NK cell therapy using autologous NK cells. NK cells are isolated from a cancer patient, expanded, and then re-infused in the same cancer patient. **(C)** Adoptive NK cell therapy using allogeneic NK cells. NK cells are isolated from a donor, expanded, and then re-infused in any recipient. The main sources for allogeneic NK cells are NK cells from peripheral blood, from umbilical cord blood, from human embryonic stem cells, from induced pluripotent stem cells and NK cell lines. **(D)** Adoptive NK cell therapy using engineered CAR-NK cells. CAR-NK cells can target and eliminate cancer cells with additional mechanisms beyond the CAR pathway, including ADCC. Figure created with BioRender.com.

Major cytokines involved in the modulation of human NK cell development, survival, and activity, include interleukins, such as IL-2, IL-12, IL-15, IL-18, and IL-21 (45) (**Figure 2**).

#### 3.1.1 IL-2

IL-2 binds to the IL-2 receptor (IL-2R). IL-2R is composed of three subunits, IL-2Rα (CD25), IL-2Rβ (CD122), and IL-2Rγ (CD132). The binding of IL-2 and IL-2Rβ and γ subunits on NK cells activates a signal transduction that induces NK cells to proliferate and eliminate their targets (46).

Treatment with IL-2 resulted in stable clinical response in patients with metastatic melanoma, renal cancer, and advanced non-Hodgkin’s lymphoma (47).

The weaknesses of therapy with IL-2 are the dose-associated toxicity (vascular leakage favoring fluid extravasation into visceral organs) and the ability to activate and expand Tregs, which in turn can induce an impairment of antitumoral activity of NK cell (47, 48). To overcome this issue, recently, an IL-2 variant has been developed. This variant (F42K) preferentially binds to IL2Rβγ subunits, expressed on NK cells, and has a decreased capacity to bind to IL-2Rα, expressed on Tregs. It has been observed that F42K upregulates the expression of NK activation markers, enhances NK-mediated cytolytic activity and is also able to induce a reduction of Tregs *in vivo* (49).

#### 3.1.2 IL-15

A valid alternative to IL-2 to boost NK cells activity is IL-15.

IL-15 can bind to IL-2Rβ and IL-2Rγ receptor expressed on T cells, NK cells, monocytes, and neutrophils. IL-15 also binds to IL-15Rα, expressed by monocytes and dendritic cells, which is a high affinity receptor for IL-15 (50).

IL-15 has similar effects to IL-2 to NK cells, including modulation of NK cell proliferation, cytotoxicity, and cytokine production (51). The advantage of using IL-15 instead of IL-2 is that IL-15 activates Tregs less consistently compared to IL-2, does not induce a significant capillary leak, and promotes prolonged expansion and activation of NK cells (52, 53).

The first in-human clinical trial employing recombinant human IL-15 (rhIL-15) in patients with metastatic melanoma and renal cell cancer (RCC) showed that administration of IL-15 is safe and that can enhance the function and proliferation of NK cells in peripheral blood of cancer patients (53). In addition, it has been reported that CD56 bright NK cells from leukemia and MM patients stimulated with IL-15 exhibited potent antitumor responses (54).

Unfortunately, the half-life of IL-15 *in vivo* is very short with a limited bioactivity of IL-15 after systemic delivery. To achieve a consistent boost of NK cells IL-15 needs to be administered continuously, but constant exposure of NK cells with IL-15 can cause their exhaustion, decreased viability, and impairment of anti-tumor activity (55, 56).

One strategy to overcome these issues was the development of stable fusion proteins, comprising IL-15 and IL-15Rα. In this regard, a promising construct is N-803 (previously named ALT-803). N-803 is a complex, consisting of an IL-15 variant (IL-15N72D) bound to an IL-15 receptorα/IgG1 Fc fusion protein. Studies *in vivo* demonstrated that N-803 has a better stability, longer persistence in lymphoid tissues, and enhanced activity against cancer compared to IL-15 (57). N-803 can increase human NK cells antitumor activity by upregulating gene expression of NK-activating receptors and factors involved in NK cells cytotoxicity, as well as by reducing expression of NK inhibitory receptors (58).

N-803 is also able to prolong human NK cells viability *in vitro* (58).

Efficacy and safety of N-803 as monotherapy or in combination with conventional antitumor agents is being evaluated in several clinical trials. Two phase I clinical trials showed that N-803 was well tolerated and achieved clinical responses in patients with hematological malignancies when used as single agent (59), and in patients with advanced NSCLC when used in combination with nivolumab (60).

Another construct with promising efficacy in enhancing NK cells antitumor activity is HCW9218. HCW9218 comprises extracellular domains of the human TGF-β receptor II and a human IL-15/IL-15 receptor α complex (61). This complex showed long-lasting biological activity *in vivo* and enhanced proliferation, cytotoxic activity, and tumor infiltration of NK cells in a syngeneic B16F10 melanoma model (61, 62).

#### 3.1.3 IL-12

Another promising cytokine to boost NK cells is IL-12. IL-12 was defined as “natural killer cell stimulatory factor” (63), since can enhance antitumor NK cells activity by increasing production of IFN-γ, stimulating cytotoxicity of activated NK cells, enhancing ADCC (64).

Although IL-12 has demonstrated promising efficacy in animal models against both solid tumors and hematologic malignancies, clinical trials did not yield convincing results (64).

One of the main reasons of poor efficacy of IL-12 in cancer patients is the accumulation of immunosuppressive cells and soluble factors in the TME, which render cancer cells resistant to the antitumor activity exerted by IL-12 (65, 66). One novel approach to increase IL-12 efficacy is the employment of NHS-IL12 immunocytokine, which is composed of two IL-12 heterodimers fused to the NHS76 antibody. The first-in-human phase I trial in subjects with metastatic solid tumors showed that NHS-IL12 increased the percentage of activated and mature NK cells and tumor-infiltrating lymphocyte density, with 5 subjects having durable stable disease (SD) (67).

#### 3.1.4 IL-18

IL-18, a member of the IL-1 superfamily, plays a role in stimulating antitumor NK cells activity. For example, IL-18 can enhance NK cells ability to kill colorectal cancer cells via the miR-574-3p/TGF-β1 axis (68) and has been proven to enhance therapeutic effects of immune checkpoint inhibitors (ICIs) against cancer cells in animal models through accumulation of pre-mNK cells, memory-type CD8(+) T cells, and suppression of Tregs (69). Despite these promising results, the employment of recombinant IL-18 had limited activity as a single agent in patients with previously untreated metastatic melanoma (70). One of the explanations of the limited clinical activity of IL-18 is because high levels of IL-18 are associated with impairment of NK cells, suggesting that IL-18 could have a double hedge sword effect in cancer immunotherapy (71, 72).

#### 3.1.5 IL-21

A similar dual role in immune-regulation has been reported for IL-21. IL-21 is a member of the cytokine family comprising IL-2, IL-4, IL-7, IL-9, and IL-15 (73, 74). IL-21 can enhance antitumor NK cells activity by upregulating NKG2D receptor expression, by inducing rapid maturation of human CD34+ cell precursors towards NK cells in cooperation with IL-7 and IL-15 (73, 74), and by potentiating ADCC (75). IL-21 showed a promising activity in stimulating NK cell cytotoxicity against several types of cancers in animal models and encouraging results in humans, especially in patients with melanoma and renal cancer (76).

IL-21 can impair antitumor activities of immune cell subsets, including inhibition of dendritic cells activation and maturation, and induction of IL-10 expression in T cells and B cells. This dual activity on the immune system raised some concerns in the employment of this cytokine in cancer immunotherapy (76).

Advantages and disadvantages to stimulate NK cells through modulatory cytokines are reported in **Table 1**.

**Table 1:** Advantages and disadvantages to stimulate NK cells through modulatory cytokines.

Modulatory cytokines	Vantages (reference)	Disadvantages (reference)	Solutions (reference)
**IL-2**	Stimulates proliferation of NK cells and induces NK cells to kill target cells (46)	Toxicity: vascular leakage favoring fluid extravasation into visceral organs (47, 48) Activates and expands Tregs (47, 48)	IL-2 variant with a decreased capacity to bind to IL-2Rα, expressed on Tregs (49)
**IL-15**	Promotes prolonged expansion and activation of NK cells (52, 53) Activates Tregs less consistently compared to IL-2 (52, 53) No significant capillary leak (52, 53)	Short half-life with a limited bioactivity after systemic delivery (55, 56) Constant exposure of NK cells with IL-15 can cause their exhaustion, decreased viability, impairment of antitumor activity (55, 56)	Stable fusion proteins, comprising IL-15 and IL-15Rα, with a better *in vivo* stability, and enhanced capacity to induce tumor infiltration of NK cells (57–62)
**IL-12**	Enhances antitumor NK cells activity by increasing production of IFN-γ, stimulating cytotoxicity of activated NK cells (64) Enhances ADCC (64)	Immunosuppressive cells and soluble factors in the TME render cancer cells resistant to IL-12 (65, 66)	NHS-IL12 immunocytokine comprising two IL-12 heterodimers fused to the NHS76 antibody. NHS-IL12 increased the percentage of activated and mature NK cells (67)
**IL-18**	Enhances antitumor NK cells activity via the miR-574- 3p/TGF-β1 axis (68) Suppression of Tregs (69)	High levels of IL-18 can cause impairment of NK cells (71, 72)	
**IL-21**	Enhances antitumor NK cells activity by upregulating NKG2D expression (73) Enhances ADCC (75)	Impairment of antitumor activities of immune cell subsets (76)	

Given the difficulties of achieving impressive clinical responses with manageable toxicities through the stimulation of NK cells with cytokines, a new strategy to improve NK cell-based immunotherapy was the development of engineered NK cells. Engineered NK cells are being used as adoptive NK cells and to enhance antibody-based immunotherapy.

### 3.2 Adoptive NK cell therapy: sources of NK cells

A promising approach to enhance NK cells antitumor activity is the adoptive cell therapy, which consists of isolating NK cells from cellular components of immune system of a subject, engineering them *ex-vivo*, expanding their numbers and then re-infuse them in a cancer patient. Sources of NK cells employed in the adoptive NK cell therapy include autologous NK cells, allogeneic NK cells, and CAR-NK cells (**Figure 2**).

#### 3.2.1 Autologous NK cells

Adoptive NK cell therapy using autologous NK cells consists of isolating NK cells from a cancer patient, expanding them *ex vivo* and then re-infusing them in the same cancer patient. One of the first applications of adoptive NK cell therapy was the employment of autologous NK cells. One example is represented by NK cells from patients with lymphoma and breast cancer that were first isolated from the blood, then stimulated overnight *ex vivo* with IL-2 and then re-infused in the same patient (77). Despite a good stimulation of autologous NK cells, their efficacy in humans seems to be modest. In this regard, one study reported that infusion with autologous NK cells stimulated and expanded *ex vivo* with heat shock protein 70 (HSP70) showed SD only in one patient out of 12 patients with refractory colorectal cancer or NSCLC (78). Similarly, no clinical response was observed in patients with unresectable, locally advanced and/or metastatic digestive cancer treated with autologous NK cells expanded *ex vivo* with OK432, IL-2, and modified FN-CH296 induced T cells (79).

The poor clinical efficacy observed with adoptive transfer of *ex vivo* activated autologous NK cells may be due to the expression of MHC class I molecules on the surface of cancer cells, which bind to inhibitory receptors on autologous NK cells. This binding can suppress the activation of NK cells. Moreover, autologous NK cells isolated from cancer patients are usually exhausted or with impaired functions. In this way, is difficult to achieve effective antitumor activity (80).

#### 3.2.2 Allogeneic NK cells

The advantage of using allogeneic NK cells versus autologous NK cells is that allogeneic NK cells can be used with any recipient. This “off the shelf” source of NK cells can overcome the labor-intensive process to obtain and stabilize the one-donor, one-patient autologous NK cells.

The main sources for allogeneic NK cells are donor peripheral blood (PB), umbilical cord blood (UCB), human embryonic stem cells (hESCs), induced pluripotent stem cells (iPSCs) and NK cell lines (81, 82).

##### 3.2.2.1 PB NK cells

The advantage to obtain and expand allogeneic PB NK cells is that allogeneic NK cells can be isolated from peripheral blood mononuclear cells (PBMCs) by CD3 depletion and subsequent CD56 enrichment to achieve high NK cells purity (83). In addition, the off-the-shelf allogeneic PB NK cells can be re-infused to the same donor to allow further product generation and donor optimization. However, allogeneic PB NK cells present some disadvantages, such as low and/or highly variable percentage of NK cells derived from a single apheresis (especially between donors), heterogeneous population of NK cells (some express more activating receptors, some more inhibitory receptors), KIR-HLA mismatch between donor and recipient which may increase the risk of getting graft versus host disease (GVHD). In this case, to avoid immunological rejection, chemotherapy is needed before the infusion of allogeneic NK cells (82, 84).

PB NK cells can also be cryopreserved, but the process of cryopreservation can alter their cytotoxic function (85).

##### 3.2.2.2 UCB NK cells

One strategy to overcome limitations of PB NK cells is to use UCB as source of NK cells. UCB has an abundance of highly cytotoxic CD56^+^ NK cells (86). NK cells derived from UCB are easy to collect, are younger and proliferate better than PB NK cells, and have less chance to trigger GVHD (87). The drawback in using UCB-derived NK cells is that they express lower levels of CD16, KIRs, IL-2R, and granzyme B as well as higher levels of inhibitory receptor NKG2A compared to PB-derived NK cells (88, 89). These features could reduce the cytotoxic activity (including mediated-ADCC) of allogeneic UCB-derived NK cells against cancer cells *in vivo* (88, 89). One strategy used to overcome this issue is to expand the UCB-derived NK cells *in vitro* using cytokines, such as IL-2, IL-12, IL-15, and IL-18 (87). Stimulation of UCB-derived NK cells with cytokines let them acquire a cytotoxicity comparable to that of PB NK cells (90).

##### 3.2.2.3 hESCs and iPSCs NK cells

A valid alternative to obtain a big source of allogeneic NK cells is represented by hESCs and iPSCs. The advantages of using NK cells derived from iPSC/hESC are the presence of homogenous population, the possibility to expand them on a large scale and the possibility to genetically modify them (91).

It has been proven that NK cells derived by hESC and iPSC are a homogeneous population of cells expressing CD56, KIRs, CD16, NKp44, NKp46, and NKG2D (92). This phenotype allows these NK cells to kill cancer cells similarly to PB NK cells (92). It has also been observed that hESC-derived NK cells have a more potent activity against cancer cells *in vivo* compared to UBC-derived NK cells (93). In addition, the possibility to modify genetically iPSCs allows to insert or delete specific genes to enhance their cytotoxicity and to have a consistent production in large scale of NK cells with identical phenotype (94).

##### 3.2.2.4 Immortalized NK cell lines

Another strategy to have a stable and homogeneous source of allogeneic NK cells was the development of immortalized NK cell lines. The most promising NK cell line that showed antitumor activity is the NK-92 cell line.

NK-92 cell line is an immortalized IL-2-dependent human NK cell line, originated from a patient with lymphoma, that lacks ADCC activity and KIRs but can kill cancer cells through perforin and granzyme B-mediated cytolytic activity (95, 96). The limitation in the use of these cells, especially in clinic, is that they require irradiation prior to infusion into the cancer patient because of the risk they can proliferate abnormally and cause lymphoma (96).

Allogeneic NK cells are widely used in clinical trials as adoptive NK cell therapy. Allogeneic NK cells were employed to treat several types of cancers including hematological malignancies and solid tumors (82). For example, the first in human study using UBC-derived NK cells in MM patients showed that UCB-derived NK cells were well tolerated (no infusional toxicity and no GVHD) and resulted in partial response (PR) in 10 patients (97). Similarly, NK-92 cells showed safety and efficacy, with two patients that achieved a complete response (CR), in a phase I trial when used to treat refractory hematological malignancies (98). Despite promising results in blood tumors, efficacy of allogeneic NK cells in solid tumor was not so high. In a phase I study, the administration of non-HLA-matched donor PB NK cells, following a lymphodepleting chemotherapy regimen, showed SD in 2 out of 6 subjects with colorectal carcinoma up to post-infusion day 100 (99). Another phase I clinical trial reported that 8 out of 17 evaluable patients with malignant lymphoma or advanced, recurrent solid tumors, showed SD after being treated with *ex vivo*-expanded and highly activated NK cells (MG4101) derived from random unrelated healthy donors (100). The lower efficacy of allogeneic NK cells in solid tumors compared to blood tumors could be due to the high immune-suppressive TME of solid tumors.

#### 3.2.3 CAR-NK cells

Chimeric antigen receptor (CAR) technology is a relatively new strategy to enhance the potency of immune system against cancer cells. This technology was initially employed to produce CAR-T cells. CAR-T cells are generated from isolated T lymphocytes from cancer patients, that are then engineered to express CARs. These modified T lymphocytes are then expanded and reinfused into the patient to achieve a stronger antitumoral response (101).

CAR-T cells achieved promising responses against hematological malignancies, but CAR-T cell therapy failed to achieve good objective responses in patients with solid tumors. This phenomenon is due to their difficulty in infiltrating solid tumors and to the capacity of TME of solid tumors to impair the activity of CAR-T cells (101–103).

In addition, there are other limitations on the use of CAR-T cells, such as their limited expansion and short persistence, high cost and intensive manufacturing process. It is important also to report the toxicity associated with CAR-T therapy, including cytokine release syndrome (CRS), neurotoxicity, and GVHD (102, 103).

To overcome these limitations, chimeric antigen receptor-engineered NK (CAR-NK) cells have been developed.

CAR-NK cells can be generated using the same CD3ζ intracellular domain used to generate CAR-T cells or involving NK-specific activating domains, such as 2B4, DAP10 and DAP12 (104).

CAR-NK cells overcome several limitations of CAR-T cells. CAR-T cells are made from autologous T cells to avoid alloreactivity and GVHD due to a mismatch of MHC molecules between the donor and the recipient. Conversely, NK cells do not require MHC pathway to be activated. This feature reduces the risk of severe alloreactive reactions and allows to use different sources of NK cells, including peripheral and cord blood NK cells, induced pluripotent stem cells (iPSCs), and NK cell lines to generate CAR-NK cells (103, 104). In addition, CAR-NK cells can be manufactured through a less intensive process compared to CAR-T cells and infused to patients at any time (103). Moreover, CAR-NK cells release different profiles of cytokines compared to CAR-T cells upon activation, thus reducing the risk to develop CRS and neurotoxicity (105).

Another advantage of using CAR-NK cells over CAR-T cells is that NK cells can target and eliminate cancer cells with additional mechanisms beyond the CAR pathway, such as ADCC and the engagement of KIRs (104).

Recently, the safety and efficacy of CAR-NK cells against hematological malignancies have been investigated by several clinical trials. Results from these studies suggest that CAR-NK cells could be used as immunotherapy for patients with multiple myeloma, lymphoma, and leukemia (105, 106).

Unfortunately, the efficacy of CAR-NK cells to treat solid tumors is still poor. This could be due to several factors, including limited tumor infiltration, limited survival and persistence in the TME, inactivation of NK cells by the immunosuppressive TME, and low CAR transduction efficiency (103).

For example, soluble factors, such as BAG-6, galectin-9, soluble NKG2D-L, cytokines, released in the TME by cancer cells, Tregs, tumor-associated macrophages (TAM) and MDSCs can result in CAR-NK cell impairment. In addition, release of indoleamine 2, 3 dioxygenase (IDO) or ROS by CAFs, as well as hypoxia and nutrient deprivation in the TME, play a role in limiting survival, activity, and persistence of CAR-NK cells in the TME (107, 108).

To solve these limitations, a novel generation of CAR-NK cells has been developed. Most promising approach include a) modified CAR-NK cells expressing chemokine receptors to improve migration and persistence of CAR-NK cells in the TME (107, 108); b) modified CAR-NK cells targeting Tregs and CAFs in TME to reduce the release of immune-suppressive cytokines and soluble factors (108); c) engineered CAR-NK cells expressing IL-12, IL-15, and IL-18 to boost NK cells antitumor activity and prolong their survival in the TME (108, 109); d) combination of CAR-NK cells with immune checkpoint inhibitors (ICIs) to prevent NK cells exhaustion and inhibition by cancer cells (107, 108). Ongoing clinical trials are evaluating the efficacy and safety of CAR-NK cells for the treatment of different locally or advanced refractory/recurrent solid tumors, such as ovarian, endometrial, testicular, pancreatic, triple negative breast, colorectal and gastric carcinoma, but results about efficacy have not yet released (104).

Advantages and disadvantages of adoptive NK cell therapy are reported in **Table 2**.

**Table 2:** Advantages and disadvantages of adoptive NK cell therapy.

Adoptive NK cell therapy	Vantages (reference)	Disadvantages (reference)	Solutions (reference)
**Autologous NK cells**	Isolated, stimulated and reinfused in the same cancer patient (no alloreactive reaction) (77)	Can be re-infused only in the same donor (labor-intensive process to obtain and stabilize the one-donor, one-patient NK cells) (80)	
	Can be stimulated with cytokines (77)	Poor clinical efficacy due to expression of MHC class I molecules on cancer cells, which bind to inhibitory receptors on autologous NK cells (80)	
		NK cells exhausted or with impaired functions (80)	
**Peripheral blood (PB) allogeneic NK cells**	Can be used with any recipient (81, 82)	Low and/or highly variable percentage of NK cells derived from a single apheresis (82, 84)	Use UCB NK cells
	Isolated from PBMCs by CD3 depletion and subsequent CD56 enrichment to achieve high NK cells purity (83)	Heterogeneous population of NK cells (82, 84)	
		KIR-HLA mismatch between donor and recipient which may increase the risk of getting GVHD (82, 84)	
	Can be re-infused to the same donor to allow further product generation and donor optimization (82, 84)	Cryopreservation can impair their cytotoxic ability (85)	
**Umbilical cord blood (UCB) allogeneic NK cells**	NK cells are easy to collect, are younger and proliferate better than PB NK cells (87)	Lower cytotoxicity activity than PB-derived NK cells (88, 89)	Expand the UCB-derived NK cells *in vitro* using cytokines to let NK cells to acquire a cytotoxicity comparable to that of PB NK cells (90)
	Have less chance to trigger GVHD (87)		
**Human embryonic stem cells (hESCs) Induced pluripotent stem cells (iPSCs)**	Homogenous population of NK cells (91)		
	Ability to be expanded on a large scale (91)		
	Possibility to be genetically modified (91)		
	More potent activity against cancer cells *in vivo* compared to UBC-derived NK cells (93)		
**Immortalized NK cell lines**	High cytotoxicity (95, 96)	Require irradiation prior to infusion because of the risk they can proliferate abnormally (96)	
**CAR-NK cells**	Lower risk of severe alloreactive reactions compared to CAR-T cells (103, 104)	Limited tumor infiltration, limited survival and persistence in the TME (103)	Modified CAR-NK cells expressing chemokine receptors (107)
	Can be generated from different sources of NK cells (103, 104)	Inactivation of NK cells by the immunosuppressive TME (103)	Modified CAR-NK cells targeting Tregs and CAFs in TME (108)
	Can be produced in large quantities and infused to cancer patients at any time (103)	Low CAR transduction efficiency (103)	Engineered CAR-NK cells expressing cytokines (108, 109)
	Can eliminate cancer cells also through ADCC (104)		

## 4 Antibody-based immunotherapy

The rationale of using antibodies for the treatment of malignant diseases was developed by Ehrlich more than a century ago (110).

Initially, antibody-based therapies employed tumor antigen allogenic or autologous polyclonal antibodies, but their use in clinic was abandoned because of their poor specificity and reproducibility.

With the development of hybridoma technology, which allowed to have tumor antigen-specific, reproducible, and immunogenic monoclonal antibodies, the employment of antibodies in the clinic became a reality (111).

Cancer cells evade immunosurveillance through several mechanisms. One of these mechanisms is the involvement of immune checkpoint pathways that allow cancer cells to inhibit antitumor immune response and to acquire a more aggressive phenotype (111–113).

During the last decades several mAbs targeting immune checkpoints pathways have been developed and approved by FDA for the treatment of human cancers.

The main pathways targeted by ICIs are cytotoxic T lymphocyte antigen-4 (CTLA-4) (112), the epidermal growth factor receptor (EGFR)-, CD20-, vascular endothelial growth factor (VEGF)-, and the programmed cell death protein-1 (PD-1)/programmed cell death protein-1 ligand (PD-L1)-mediated pathway (111).

The PD-1/PD-L1 checkpoint pathway represented one of the most targeted pathways by monoclonal antibodies used in clinic. The binding between PD-L1, over-expressed on cancer cells, and PD-1, expressed on activated T-cells, leads to cancer cells inhibiting the function of the cytotoxic T cells in the TME, and evading the pressure of the immune system. Anti–PD-1/PD-L1 mAbs are able to block the binding between PD-1 and PD-L1 to revert immunosuppressive signals by cancer cells and to enhance immune cells activity in the elimination of malignant cells (113).

Among them, nivolumab, pembrolizumab, atezolizumab, durvalumab, have demonstrated clinical efficacy against several tumor types.

Nivolumab, a fully human IgG4-blocking mAb directed against PD-1, produced objective responses ranging from 13.3 to 40% in patients with advanced melanoma (114), renal-cell (115), NSCLC (116), colorectal (117), head and neck (118), urothelial cancer (119).

The anti PD-1 mAb pembrolizumab has shown potent antitumor activity in patients with different solid tumors. Pembrolizumab provided substantial benefits in overall survival (OS), progression free survival (PFS), and overall response rate (ORR) in participants with NSCLC (120, 121), HNSCC (122), uterine (123), cervical (124), urothelial (125), Merkel-cell carcinoma (126), and melanoma (127).

A very promising clinical efficacy against PD-L1 expressing tumors has also been proved using anti-PD-L1 mAbs atezolizumab and durvalumab. For example, atezolizumab demonstrated a promising antitumor activity in patients with metastatic renal cell carcinoma, where an increased T-cell-to-regulatory T-cell ratio correlated with a better response to therapy (128, 129). In locally advanced and metastatic urothelial and in NSCLC patients, treatment with atezolizumab resulted in a significantly improved objective response rate (130–133). In addition, patients with metastatic triple-negative breast cancer with a higher PD-L1 expression showed a better clinical response than patients with a lower PD-L1 expression after treatment with atezolizumab in combination with nab-paclitaxel (134, 135). Similar results were achieved with durvalumab (136).

### 4.1 FDA approved mAbs with ADCC activity: the role of NK cells and of CD16 polymorphisms

Although the employment of ICIs as immunotherapeutics has improved survival of cancer patients, not all patients achieved a good clinical response. This phenomenon can be due partially to tumor resistance to ICIs. Cancer cells, at level of TME, developed resistance to ICIs through reduction of expression of tumor antigens, uncontrolled activation of oncogenes, increased activity of Tregs and MDSCs (137, 138).

To overcome the resistance to ICIs, mAbs were engineered to kill cancer cells through ADCC mediated by NK cells.

The advantage of using mAbs with ADCC activity is that they can activate NK cells, but also stimulate the secretion of cytokines, which in turn facilitates the accumulation of immune effector cells to the TME (26). As already mentioned, ADCC is a mechanism that needs the binding of the Fc portion of mAbs to CD16 expressed on NK cells (26).

CD16 can have three different genotypes based on the presence of valine (V) or phenylalanine (F) at amino acid 158. Each genotype (V/V, V/F, F/F) confers to NK cells a different affinity for the Fc portion of mAbs, which in turn can result in a better or worse clinical outcome in cancer patients treated with mAbs with ADCC activity (139).

FDA approved several mAbs that can mediate ADCC. A correlation between NK cells CD16 genotype and clinical response has been observed in cancer patients treated with trastuzumab, rituximab, cetuximab, and avelumab.

#### 4.1.1 Trastuzumab

Trastuzumab is a mAb which targets the human epidermal growth factor receptor 2 (HER2). HER2 is a member of the ErbB family that plays an important role in promoting oncogenic transformation and tumor growth. Overexpression of HER2 has been found in breast, ovarian, bladder, salivary gland, endometrial, pancreatic and NSCLC, and is usually linked with a poor prognosis (140).

Trastuzumab was approved by the FDA as a biological approach for the treatment of metastatic breast cancer in 1998 (141). FDA has also approved trastuzumab in combination with chemotherapy for HER2-positive metastatic cancer of the stomach or gastroesophageal junction in 2010 (142).

A pilot trial conducted in patients with operable breast tumors overexpressing HER2 demonstrated that trastuzumab was able to mediate ADCC in 15 of 18 patients (83%). Moreover, a patient which had a high ADCC activity showed a complete pathologic response, while patients unable to mount ADCC did not have a significant tumor regression after therapy. These data suggested that the response of breast cancer patients to trastuzumab may be due in part to the involvement of ADCC mechanism (143).

Different studies also showed that NK cells CD16 polymorphisms resulted in improvement of clinical outcomes in cancer patients treated with trastuzumab.

Musolino et al. demonstrated that patients with metastatic breast cancer expressing HER-2/neu and harboring the CD16 158 V/V genotype had a significantly higher trastuzumab-mediated cytotoxicity compared to other two genotypes and that patients with V/V genotype achieved a better ORR and PFS (144).

The tendency of the V/V genotype to be correlated with the objective response in metastatic HER2-positive breast cancer patients treated with trastuzumab has also been confirmed in another study by Tamura et al. (145).

#### 4.1.2 Rituximab

Rituximab is a genetically engineered chimeric (murine-human) mAb which recognizes the B-cell-specific antigen CD20 expressed on various lymphoid malignancies, such as non-Hodgkin’s lymphomas (NHL) and B-cell chronic lymphocytic leukemia (CLL) (146, 147).

This mAb was approved by FDA for the treatment of relapsed low-grade or follicular B-cell non-Hodgkin’s lymphoma in 1997 (146), for the treatment of chronic lymphocytic leukemia in combination with fludarabine and cyclophosphamide in 2010 (148), and for the treatment of adult patients with Waldenström’s macroglobulinemia in 2018 (149).

As for trastuzumab, the CD16 158 V/V genotype resulted in an improved outcome also in patients treated with rituximab.

For example, patients with follicular lymphoma harboring CD16 V/V genotype had an improved clinical response to rituximab (150).

Similarly, Veeramani et al. suggested that the superior clinical outcome seen in patients with lymphoma harboring high affinity CD16 158 genotypes (V/F or V/V), compared to the low-affinity genotype (F/F), may be due to a quicker NK cells activation in subjects with the CD16 V/V genotype after treatment with rituximab (151).

The V/V genotype was also significantly correlated with a higher CR rate to rituximab plus cyclophosphamide/doxorubicin/vincristine/prednisone (R-CHOP) therapy in diffuse large B-cell lymphoma (DLBCL) compared with the F/F genotype (88% in V/V vs 50% in F/F) (152).

In a study conducted on patients affected by Waldenström’s macroglobulinemia, Treon et al. observed that the response trend to rituximab was significantly higher for patients with the V/V genotype compared to patients with the F/F genotype (40.0% vs 9%) (153).

#### 4.1.3 Cetuximab

Cetuximab is a chimeric immunoglobulin G1 (IgG1) which targets EGFR, a member of receptor tyrosine kinases (TK) highly expressed in several types of cancers, including breast, lung, colorectal and head and neck cancers. The binding between EGFR and its ligands activates a downstream signaling cascade that in turn promotes proliferation, differentiation, migration, and survival of cancer cells (154).

Cetuximab received FDA approval to treat patients with metastatic advanced colorectal cancer in 2004 (155). In 2011, cetuximab received FDA approval in combination with cisplatin or carboplatin and 5-fluorouracil for the first-line treatment of HNSCC (156).

In a study performed in patients with locally advanced head and neck cancer treated with cetuximab and radiotherapy, Lattanzio et al. observed that patients with elevated basal ADCC and high EGFR expression had a better chance of achieving a CR and a long OS compared to the others, suggesting a possible important role of ADCC in predicting favorable outcome (157).

Even for cetuximab it has been proved that the CD16 158 V/V genotype plays an important role in enhancing the ADCC.

In patients with metastatic colorectal cancer with KRAS mutations treated with cetuximab plus irinotecan it has been demonstrated that V/V genotype carriers had a longer PFS than F/F genotype carriers (5.5 v 3.0 months). Since patients with KRAS mutations were found to have a lower response rate and a shorter PFS than patients with non-mutated KRAS, the fact that the V/V genotype was correlated with a better PFS suggests an important role of the CD16 158 polymorfism in the ADCC activity and clinical outcome of patients treated with cetuximab (158).

Trotta et al. also demonstrated that colorectal cancer patients with the V/V genotype showed a significantly higher ADCC mediated by cetuximab and a significantly longer PFS than patients with F/F genotype (159).

Similarly, NK cells expressing the V/V genotype were found to be significantly more effective than those expressing V/F and F/F genotypes in mediating ADCC against HNSCC in presence of cetuximab, supporting the importance of the CD16 158 polymorphism in patient variability of cetuximab mediated clinical responses (160, 161).

#### 4.1.4 Avelumab

Avelumab is a fully human IgG1 targeting PD-L1 on cancer cells.

Avelumab has been proved to mediate ADCC against a range of human cancer cells, including lung, breast, and bladder carcinomas (162–164). A preclinical study showed that subjects with the CD16 158 V/V genotype had higher capacity to kill cancer cells through ADCC mediated by avelumab than subjects with the F/F genotype (162).

Avelumab received FDA approval for the treatment of metastatic Merkel cell carcinoma (mMCC) in adults and pediatric patients aged ≥12 years and for the treatment of urothelial carcinoma in 2017 (165, 166). In 2019, avelumab received FDA approval in combination with axitinib for the treatment of patients with advanced renal cell carcinoma (167). Even if clinical studies to identify a link between CD16 polymorphism and better clinical outcome after treatment with avelumab are still ongoing, the ADCC mediated by avelumab resulted in clinical responses in several clinical trials.

In this regard, a multicenter, single-group, open-label, phase II trial showed that avelumab was associated with durable responses and was well tolerated in patients with chemotherapy-refractory metastatic Merkel cell carcinoma (168).

In a phase 3 trial, conducted in patients with advanced renal-cell carcinoma, the combination of avelumab and axitinib, a VEGF pathway inhibitor, resulted in an enhanced clinical benefit. Among patients with PD-L1-positive tumors, the ORR was 55.2% and the median PFS was 13.8 months when these agents were administered as first-line treatment (169). In a recent clinical trial, combining avelumab and cetuximab as second- or third-line treatment in patients with NSCLC, it has been proved that the clinical benefit of the combination of these two mAbs was determined by NK cell-mediated ADCC (170).

## 5 Engineered NK cells to enhance mAbs-mediated ADCC

**Figure 3:**
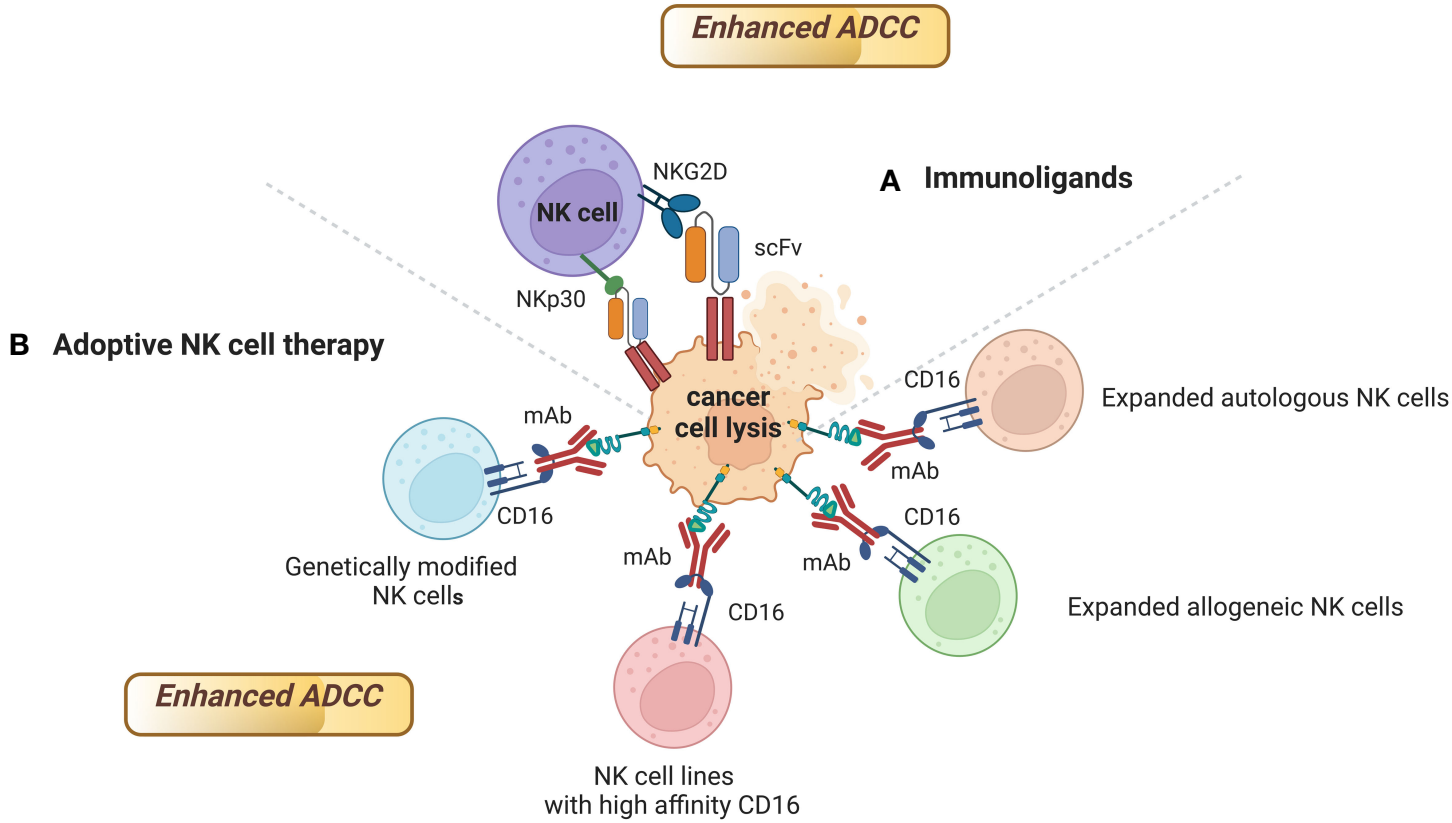
Strategies to enhance FDA approved mAbs-mediated ADCC using engineered NK cells as effectors. mAbs able to mediate ADCC can activate NK cells, but also stimulate the accumulation of immune effector cells to the tumor site. Engineered NK cells have been found to enhance ADCC activity of several FDA approved mAbs. Sources of engineered NK cells as effectors include **(A)** Immunoligands; **(B)** Expanded autologous or allogeneic NK cells, NK cell lines with high affinity CD16, genetically modified NK cells. Figure created with BioRender.com.

Several strategies have been developed to enhance FDA approved mAbs-mediated ADCC using engineered NK cells as effectors, including immunoligands, and adoptive NK cell therapy (**Figure 3**).

### 5.1 Immunoligands

One example of the importance of immunoligands to enhance mAbs-mediated ADCC is the development of immunoligands fusing the extracellular domains of ligands of the activating NK cell receptors NKp30, NKp80, DNAM-1 and NKG2D to a single-chain fragment variable (scFv) targeting HER2 on different solid tumors (171, 172). The binding of these immunoligands to HER2 on cancer cells and to NK cell receptors enhanced ADCC mediated by trastuzumab and cetuximab against HER2 positive cancer cells (171, 172) (**Table 3**).

**Table 3:** Efficacy of engineered NK cells in enhancing FDA approved mAbs-mediated ADCC.

Engineered NK cells	Combination	Study	Efficacy	Reference
**Immunoligands**	Immunoligands fusing the extracellular domains of ligands of the activating NK cell receptors to a scFv targeting HER2 on different solid tumors	Preclinical	Enhanced ADCC mediated by trastuzumab and cetuximab against HER2 positive cancer cells	(171, 172)
	Rituximab with CD20-specific immunoligands targeting NKG2D	Preclinical	Enhanced ADCC mediated by rituximab against B-chronic lymphocytic leukaemia and Burkitt's lymphoma cell lines	(173)
**Expanded *ex vivo* NK cells**	Expanded NK cells in combination with trastuzumab and cetuximab	Phase I in patients with gastric or colorectal cancer	Four patients achieved SD. Reduction of tumor size in three patients with SD	(174)
	Trastuzumab in combination with autologous NK cells expanded with K562-mb15-41BBL cells	Phase I in patients with treatment-refractory HER2-positive solid tumors	Six patients achieved SD for ≥6 months	(175)
	Expanded autologous NK cells with IL-15 in combination with rituximab	Phase I in patients with relapsed CD20-positive malignant lymphoma	NK cells enhanced ADCC mediated by rituximab. Seven patients maintained CR with a median duration of 44 months	(176)
	Expanded allogeneic NK cells in combination with rituximab	Phase I in patients with relapsed/refractory B cell non-Hodgkin lymphoma	PR in 4 patients and CR in 1 patient, with an overall response rate of 55.6%	(177)
	NK cells stimulated with nicotamide in combination with rituximab	Phase I in patients with advanced non-Hodgkin lymphoma	ORR of 74% in 19 patients, CR in 13 patients, PR in 1 patient	(178)
	Expanded NK cells in combination with cetuximab	Phase I/II in patients with NSCLC	Longer PFS, reduction of levels of circulating cancer cells compared to patients treated with cetuximab alone	(179)
	Autologous NK cells, expanded by co-culture with irradiated K562-mb15-41BBL cells in combination with cetuximab	Phase I in patients with recurrent/metastatic nasopharyngeal carcinoma	SD in 4 out of 7 patients	(180)
**Immortalized NK cell lines**	haNK cell line in combination with trastuzumab, cetuximab and avelumab	Preclinical	haNK potentiated *in vitro* ADCC activity of mAbs	(181–183)
	NK92-41BB cell line harboring CD16/CAR with high affinity for Fc portion of mAbs in combination with trastuzumab and rituximab	Preclinical	Enhanced ADCC activity	(184)
	Endogenous CD16-expressing NK cell line (oNK) conjugated with trastuzumab	Preclinical	Enhanced cytotoxicity against cancer cells expressing HER2	(185)

Similarly, it has been observed that the combination of rituximab with CD20-specific immunoligands targeting NKG2D can improve rituximab-mediated ADCC by NK cells against lymphoma (173) (**Table 3**).

### 5.2 Adoptive NK cell therapy

The employment of adoptive NK cell therapy to enhance ADCC of mAbs is another promising strategy (**Table 3**). Sources of NK cells with a pre-clinical or clinical efficacy in enhancing ADCC of mAbs include expanded autologous or allogeneic NK cells, NK cell lines, genetically modified NK cells.

#### 5.2.1 Expanded autologous or allogeneic NK cells

Recently, a phase I clinical trial evaluated the safety and efficacy of adoptive transfer of expanded NK cells in combination with trastuzumab and cetuximab in patients with gastric or colorectal cancer. Expanded NK cells with high expression of NKG2D and CD16 were infused in cancer patients after three days of mAbs administration. The combination was well tolerated and, among six evaluable patients, four patients achieved SD and two progressed. The treatment resulted also in a reduction of tumor size in three out of four patients with SD and this phenomenon was suggested to be linked to an enhanced whole blood IFN-γ production and reduction of Tregs (174).

Similarly, another phase I clinical trials showed that 6 of 19 patients with treatment-refractory HER2-positive solid tumors achieved SD for ≥6 months after receiving the combination of trastuzumab and autologous NK cells expanded by 10-day co-culture with K562-mb15-41BBL cells. These results support the rationale to use this combination in phase II trials (175).

A phase I study of cellular therapy for the treatment of patients with relapsed CD20-positive malignant lymphoma showed that NK cells, *ex vivo* expanded with IL-15 and then re-infused intravenously one day after rituximab administration, enhanced the ADCC mediated by rituximab. Seven of the nine patients maintained CR with a median duration of 44 months after NK-cell infusion. It is important to note that eight of the nine patients received chemotherapy after NK-cell infusion, rendering difficult to understand the exact contribution of the expanded NK cells to the clinical response (176). Furthermore, in a recent phase I clinical trial it has been evaluated safety and efficacy of *ex vivo*-expanded allogeneic NK cells (MG4101) in combination with rituximab for the treatment of relapsed/refractory B cell non-Hodgkin lymphoma. Results from the trial showed that the combined therapy was safe and resulted in PR in 4 patients and CR in 1 patient, with an ORR of 55.6% (177). In addition, two patients achieved prolonged responses and low exhaustion marker levels in T cells (177).

Similarly, a phase I clinical trial combining NK cells stimulated with NAM (GDA-201) and rituximab showed that the combination was well tolerated and yielded an ORR of 74% in 19 patients with advanced non-Hodgkin lymphoma (CR in 13 patients and PR in 1 patient) (178).

Another phase I clinical trial documented objective clinical responses in 3 out of 9 patients with liver metastasis from colorectal or pancreatic cancers after delivery of allogeneic NK cells in the liver combined with intravenous infusion of cetuximab and subcutaneous administration of IL-2 (186).

Adoptive NK cell therapy in combination with cetuximab also demonstrated to prolong survival in patients with advanced NSCLC. Liang et al. reported that patients treated with NK cell therapy + cetuximab had a significant improvement of quality of life and of the function of immune system. These features were associated with a longer PFS and a significantly reduction of levels of circulating cancer cells compared to patients treated with cetuximab alone, suggesting that the adoptive NK cell therapy can enhance the efficacy of cetuximab for the treatment of NSCLC (179).

A promising clinical response (SD in 4 out of 7 patients) was also observed in patients with nasopharyngeal carcinoma treated with a combination of autologous NK cells, expanded by co-culture with irradiated K562-mb15-41BBL cells, and cetuximab (180).

#### 5.2.2 NK cell lines or genetically modified NK cells

Enhancement of the ADCC activity can also be achieved using engineered NK cell lines or genetically modified NK cells (**Table 3**).

One example is represented by the engineered NK cell line haNK. The haNK cell line consists of the NK-92 cell line engineered to express CD16 with the high affinity (ha) genotype V/V, as well as to express IL-2. In this case, this NK cell line has the double advantage to mediate ADCC and to lyse cancer cells through an enhanced release of perforin and granzyme due to the constant expression of IL-2 (181). HaNK showed the ability to potentiate *in vitro* the ADCC activity of several clinical approved mAbs, including trastuzumab, cetuximab and avelumab (181–183). In addition, killing of cancer cells by irradiated haNK cells through ADCC mediated by avelumab was also shown to be similar to the killing achieved by NK cells bearing the CD16 V/V genotype (183). Another NK cell line (NK92-41BB), able to express high levels of granzyme and engineered to express chimeric genes CD16/CAR with high affinity for Fc portion of mAbs, showed a strong ADCC activity when tested in combination with trastuzumab and rituximab (184).

NK cell lines can also be generated using the Antibody-Cell Conjugation (ACC) technology. ACC provides an efficient platform to arm NK cells with antibodies targeting cancer cells to bind and kill malignant tumors. Li et al. established an NK cell line (oNK) expressing endogenous CD16 and conjugated oNK with trastuzumab and an anti-human HER2 antibody using an ACC platform. This trastuzumab-conjugated oNK, designated as ACE1702, exerted *in vitro* and *in vivo* antitumor activity against cancer cells expressing HER2, suggesting that it could be used as a novel NK cell therapy against solid tumors that express HER2 on their surface (185).

Promising results with immortalized NK cell lines provide the rationale for genetically modifying NK cells to enhance the ADCC activity of IgG1 antitumor mAbs.

Genetically modified NK cells in combination with mAbs with ADCC activity could represent a new promising strategy to achieve good clinical outcomes in several types of tumors. For example, Carlsten et al. developed genetically modified NK cells using the mRNA technology. In their study, authors demonstrated that NK cells genetically modified through electroporation with mRNA coding for CD16 158 V/V genotype potentiated NK cells cytotoxicity against lymphoma cells coated with rituximab (187). These results suggest that this approach can be utilized to improve the efficacy of adoptive NK cell-based cancer immunotherapies in combination with mAbs with ADCC activity (187).

## 6 Conclusion & future perspectives

The concept of harnessing the immune system against cancer opened the era of cancer immunotherapy for the treatment of solid and liquid cancers. The advantage of immunotherapy over chemotherapy or radiotherapy is the possibility to redirect cellular components of immune system to attack selectively cancer cells, minimizing toxicity of the treatment. Among cellular components of immune system, NK cells play a fundamental role in controlling tumor growth. NK cells can target and kill cancer cells through NK-direct killing, a mechanism that involve NK cells only, or through ADCC, a mechanism that allows NK cells to target and eliminate cancer cells via binding to an antibody already bound to a specific antigen expressed by cancer cells. Cancer cells have developed several mechanisms to evade the antitumor activity of NK cells. Immunotherapy led to the development of several strategies to enhance the antitumor activity of NK cells against cancer cells, with the goal of overcoming cancer cells resistance. In this regard, the three main strategies to engineer and boost NK cells include boosting NK cells with modulatory cytokines, adoptive NK cell therapy, and the employment of engineered NK cells to enhance antibody-based immunotherapy.

Boosting NK cells with cytokines provided the opportunity to achieve superior antitumor activity of NK cells against several types of cancers, but this strategy has the problem of systemic toxicity of cytokines, as well as the possibility to enhance proliferation of immune-suppressive cells.

Given the difficulties of achieving impressive clinical responses with manageable toxicities through the stimulation of NK cells with cytokines, NK cell-based immunotherapy was improved through the development of engineered NK cells. Engineered NK cells are being used as adoptive NK cells and to enhance antibody-based immunotherapy.

One of the first applications of adoptive NK cell therapy was the employment of autologous NK cells, which consist of isolating NK cells from a cancer patient, stimulating them *ex vivo*, and then reinfusing them in the same patient. Although the stimulation of autologous NK cells *ex vivo* can be satisfactory, their efficacy in humans seems to be modest.

The poor clinical efficacy may be due to the expression of MHC class I molecules on the surface of cancer cells, which bind to inhibitory receptors on autologous NK cells. This binding can suppress the activation of NK cells. In addition, autologous NK cells isolated from patients with malignant tumors usually are exhausted or with impaired functions (80).

To overcome this problem, the new strategy was to develop allogeneic NK cells.

The advantage of using allogeneic NK cells versus autologous NK cells is that allogeneic NK cells can be used with any recipient, thus overcoming the labor-intensive process to obtain and stabilize the one-donor, one-patient autologous NK cells (81, 82).

Allogeneic NK cells are widely used in clinical trials to treat hematological malignancies and solid tumors (82). Despite promising results in blood tumors, efficacy of allogeneic NK cells in solid tumor was modest. One reason for the lower efficacy of allogeneic NK cells in solid tumors could be due to the high immune-suppressive TME of solid tumors. A new strategy to create engineered NK cells targeting cancer cells in a more selective and aggressive manner was the development of CAR-NK cells.

The advantage of using CAR-NK cells is the fact that CAR-NK cells have a low risk of severe alloreactive reactions, can be generated by different sources of NK cells, can be produced in big quantity, and can be infused to cancer patients at any time (103).

Another advantage of using CAR-NK cells is that NK cells can target and eliminate cancer cells with additional mechanisms beyond the CAR pathway, such as ADCC and the engagement of KIRs (104).

Although CAR-NK cells achieved promising clinical responses in hematological malignancies, the efficacy of CAR-NK cells to treat solid tumors is still poor. This could be due to lack of tumor infiltration, limited persistence in the TME, inactivation of NK cells by TME (103). To solve these limitations, a novel generation of CAR-NK cells has been developed, including modified CAR-NK cells expressing chemokine receptors (107), modified CAR-NK cells targeting Tregs and CAFs in TME (108), engineered CAR-NK cells expressing IL-12, IL-15, and IL-18 (108, 109), combination of CAR-NK cells with ICIs (107, 108).

Due to the ability of cancer cells to resist to NK cells direct killing, a novel approach was to develop engineered NK cells to enhance the ADCC activity of mAbs approved for the treatment of several cancers.

Several strategies have been developed to enhance FDA approved mAbs-mediated ADCC using engineered NK cells as effectors, including immunoligands, adoptive NK cell therapy, NK cell lines, genetically modified NK cells. Several clinical trials reported that the combination of engineering NK cells with mAbs with ADCC activity can result in a superior clinical response compared to mAbs alone.

To make this combination more effective and potent, future perspectives are to further engineer both mAbs and NK cells. One strategy that is currently being implemented is to increase the affinity of mAbs to CD16 to enhance their ADCC activity.

These modifications can be done by altering the mAb Fc portion through different methodologies, such as site-directed mutagenesis, altering Fc domain glycosylation and/or taking out fucosylation of the Fc domain. In this regard, several mAbs with modified Fc portion have already shown enhanced activity compared to unmodified mAbs (188).

Some of these engineered mAbs, such as margetuximab, mogamulizumab, obinutuzumab, tafasitamab, have recently obtained approval for the treatment of several cancers (189–192).

Other strategies could be the employment of bi-specific antibodies, bi-specific killer cell engagers (BIKEs), combinations of NK cells with mAbs able to recognize and eliminate immunosuppressive cells in the TME.

For example, Pan and colleagues developed a bi-specific fusion protein (mAb04-MICA) comprising an antibody targeting vascular endothelial growth factor receptor 2 (VEGFR2) fused to a MICA α1-α2 ectodomain. Authors observed that mAb04-MICA had a better antitumor efficacy in mice bearing gastric cancer and NSCLC compared with the anti-VEGFR2 mAb ramucirumab (193).

Cancer cells can develop resistance to ADCC mediated by mAbs by inhibiting the activation and function of NK cells in the TME. One strategy to overcome this resistance is the development of BIKEs.

BiKEs concept is promoting the infiltration of NK cells to tumor site, bringing them close to cancer cells. BIKEs consist of individual heavy (VH) and light (VL) chains of the variable region of each different antibody (scFv) specific for CD16, NKG2D, NKp30 on NK cells and for tumor-associated antigens on cancer cells. In this way, NK cells can target easier cancer cells and once in close contact with them are facilitated to form cytolytic synapses that allow them to release granzymes and perforins to kill cancer cells (194). Since the antitumor activity of NK cells can also be impaired by immunosuppressive cells in the TME, the employment of mAbs able to recognize and eliminate immune-suppressive cells in the TME has the advantage of reducing NK cells impairment and exhaustion. Combining engineered NK cells with these mAbs would allow NK cells to remain in an activated status and to kill cancer cells resistant to NK cells direct killing or to ADCC mediated by mAbs.

These strategies are currently under preclinical investigation and if they achieve promising antitumor activity they could be used as rationale for the next generation of clinical trials with the scope to provide more effective and higher-quality treatments to cancer patients.

Another important point to take into consideration using NK cell therapy for treatment of cancer is the off-the-shelf applicability. NK cells can be expanded *ex-vivo* in large quantity using co-culture of NK cells with irradiated feeder cells, such as RPMI8866, EBV-LCL, and K562.

These feeder cells promote expansion of NK cells by presenting them activating ligands. In addition, to expand NK cells population over the time, NK cells can be periodically restimulated by feeder cells *ex vivo*. This expansion can be made in seed bioreactors which can allow to produce up to 10^11^ to 10^12^ cells of highly active NK cells ready to be infused to several patients at the same time. Since it is unclear if the expansion of NK cells through feeder cells can be sustained for long periods, feeder-cell free activation methods to expand NK cells are in development phase to obtain large number of stable activated NK cells with lower costs of biomanufacturing (195).

## Author contributions

MF: Conceptualization, Data curation, Writing – original draft. PA: Funding acquisition, Visualization, Writing – review & editing. KT: Conceptualization, Data curation, Supervision, Visualization, Writing – review & editing.
